# Relationship Between Mean Faecal Gastrointestinal Nematode Egg Excretion in Horses and Its Variability: Implications for Control

**DOI:** 10.3390/pathogens15020156

**Published:** 2026-02-02

**Authors:** Jacques Cabaret, Cristina Guerrero Molina, Cintli Martínez-Ortiz-de Montellano, Yazmin Alcala Canto

**Affiliations:** 1 SantéSocioVéto, 8 Pl. Carré de Busserolle, 37100 Tours, France; 2 Departamento de Parasitologia, Facultad de Medicina Veterinaria y Zootecnia, Universidad Nacional Autonoma de Mexico (UNAM), Ciudad de Mexico 04510, Mexico; mcgromo@hotmail.com (C.G.M.); cintli@unam.mx (C.M.-O.-d.M.); yazmin@unam.mx (Y.A.C.)

**Keywords:** cyathostomins, nematode, faecal egg count, horse, mean, variance

## Abstract

Faecal egg counts (FECs) are used to assess the intensity of gastrointestinal nematode (GIN) infections in herbivores. FEC distribution is aggregated, meaning that approximately 20% of animals harbour 80% of infections. In times of escalating anthelmintic resistance, it may be necessary to restrict treatment to the animals with the heaviest infections. This strategy is called targeted selective treatment (TST) and is relevant to GIN, for example. The difficulty lies in identifying which animals to treat. One solution is to select potentially at-risk animals based on age (for example, treating the young) or to perform individual faecal egg counts (though this is costly). We propose a solution for determining the suitability of selective treatment based on the level of FEC (200 or 500 eggs per gram of faeces). First, we demonstrated that the mean FEC in a group is strictly related to its variance (Taylor’s power law) using published data and our own unpublished data on horses from France, Poland, and Mexico. The study focused on small and large strongyles in horses. Taylor’s power law states that sample variance (Var) and the population mean are related by a simple equation: Var = a Mean^b or log(Var) = log(a) + b log(Mean). The influence of factors such as age, status (mare, stallion, yearling, etc.), day-to-day variability, and previous anthelmintic treatments did not alter this relationship. To reduce the number of FECs, we estimated the mean FEC on a composite faecal sample. We then calculated the variability and therefore the number of horses with an FEC above the chosen acceptable level. When the mean is high, the number of horses to be treated is also high and TST is not beneficial. When the FEC is average, TST may be worthwhile, either based on the FEC of individual horses or on the horse class at risk. Based on the percentage of horses with an FEC above the acceptable level, farmers can decide whether to treat all animals or establish a TST protocol. Caution should be exercised when using TST in the presence of large strongyles.

## 1. Introduction

The aggregation of parasites among hosts is almost universal [1]. This means that their distribution is overdispersed (or aggregated). For example, 20% of sheep concentrate 80% of gastrointestinal nematodes (GINs) [2], and 20% of horses concentrate 80% of GINs [3,4]. Several measures of aggregation have been adopted, but they actually measure different things. The variance-to-mean ratio and the k index of aggregation are the most commonly used for a single host–parasite population [5]. Variance-to-mean is positively associated with mean, whereas k appears completely uncorrelated with mean; both are somewhat positively correlated with prevalence [5]. The k index of aggregation has been evaluated in GIN in sheep [6,7], among others. When considering several host-parasite populations, the Taylor power law (TPL) has been proposed [8]. The TPL states that the variance (Var) of a parameter and its mean in a population are related by a simple power law. This can be written as Var = a Mean^b or log(Var) = log(a) + b log(Mean), where b is an indicator of aggregation. This law was initially applied in agronomy [9] but has since been extended to various biological and non-biological systems [10]. The power law and aggregation have been studied in gastrointestinal nematodes of sheep [6,7] and in sheep, cattle, goats, and deer [11]. These studies were based on necropsies, with only one based on the faecal egg counts of two species of sheep gastrointestinal nematodes (GINs) [11]. To our knowledge, neither adult GIN load nor faecal egg counts (FECs) in horses have been studied. FECs represent an important tool for managing GIN infections in horses. High infection rates are detrimental to horses [12,13], and repeated anthelmintic use has resulted in GIN resistance [14,15]. As with the distribution of adult worms, the distribution of FECs is aggregated [16], and TPL could be employed. In times of escalating anthelmintic resistance, it may be necessary to restrict treatment to the most heavily infected animals. This strategy is called targeted selective treatment (TST) and applies to GINs. This approach has long been proposed for horses [17,18], recommended [19,20], and implemented [21,22,23,24]. The technical difficulty lies in identifying the animals to be treated. Horse breeders are reluctant to undertake individual faecal egg counts (FECs) since the cost of anthelmintics is relatively low [25]. Their intention to use FECs prior to deworming is not influenced by the perceived risk of anthelmintic resistance or gastrointestinal nematode infection; rather, it depends mostly on the opinion of their peers and social pressure [26]. These beliefs may reduce the use of FECs and hence the development of TST. One solution is to select animals at risk based on age (for example, treating the young), and another is to perform individual FECs, though this is costly. We propose a solution for determining the suitability of selective treatment based on the level of accepted FECs (200 or 500 eggs per gram of faeces) and TPL. First, we evaluate the relationship between the mean FEC and its variance using Taylor’s Power Law. Published and unpublished data on horses from France, Poland, and Mexico were used. The studies concerned populations with small and large strongyles. We then evaluate the influence of factors such as age, status (mare, stallion, yearling, etc.), day-to-day variability, and previous anthelmintic treatments on the TPL parameters. Additionally, to reduce the number of individual FECs, we estimate the mean FEC on a composite faecal sample. We then calculate the variability from the mean and therefore determine the number of horses with an FEC above the chosen acceptable level. When the mean FEC is high, the number of horses to be treated is also high and TST is not beneficial. When the FEC is average, TST may be worthwhile based either on individual FEC or on the horse class at risk.

## 2. Materials and Methods

### 2.1. Characteristics of Sampling Sites

The characteristics are presented in Table 1. Some data are extracted from pre-existing studies (Poland [27] and its database or France 2 [28]), and others are our new data.

### 2.2. Parasitological Methods

The FECs were assessed with a McMaster technique using salt solution (specific gravity 1.20) with a sensitivity of 50 nematodes eggs per gramme of faeces. There were slight differences in procedure according to the sites (quantity of faeces, 3 or 5 g; additional floatation for zero counts with McMaster method). The larval cultures were performed at all sites according to [30]. The identification (small or large strongyles) was on morphological features (number of intestinal cells, length of the larvae) as presented in [31] on several occasions and on 100 larvae each time (Table 1). The following main species of cyathostomins were identified on third-stage larvae in Poland: *Cylicocyclus nassatus*, *Cylicostephanus goldi*, *Cyathostomum catinatum*, *Cylicostephanus longibursatus*, *Cyathostomum pateratum*, *Cylicocyclus ashworti*, *Cylicocyclus insigne*, and *Cylicostephanus calicatus.* The main species of large strongyles was *S. vulgaris* in Poland. In Mexico, the following *Strongylus* species were found *S. edentatus*, *S. equinus*, and *S. vulgaris*. Their respective proportions based on third-stage larvae monitored on six occasions were as follows: 16, 24, 7% Mexico 1; 14, 28, 12% Mexico 2; 15, 29, 14% Mexico 3; and 9, 12, 14 Mexico 4.

The composite faecal samples were obtained from mixing 10 individual faecal samples, and 10 FEC tests were performed. The composite established on ground samples in the stable was established as follows: 20 fresh half faecal drops from different piles were mixed and processed as for the other composite to perform 10 FEC evaluations. The efficacy of used anthelmintics was evaluated by FEC tests at treatment and 14 days after on at least 10 horses or ponies at all sites except Poland according to [31].

### 2.3. Statistical Methods

We used a linear regression method to relate variance to mean or individual versus composite EPG (eggs of gastrointestinal nematodes per gramme of faeces) and considered a statistical value of *p* < 0.05 as significant. To evaluate the stability of the EPG in faeces, 47 adult Welsh ponies (France 1) were individually sampled and their EPG measured on two consecutive days. The EPGs of each day were bootstrapped for 10 animals and their means and variances calculated. Non-linear fit was performed when linearity was not found, and the choice of the fit was then achieved based on the Akaike criterion.

## 3. Results

### 3.1. Relationship Between Average FEC and Its Variance: Factors of Variation

#### 3.1.1. Sampling Day on a Farm

**Figure 1:**
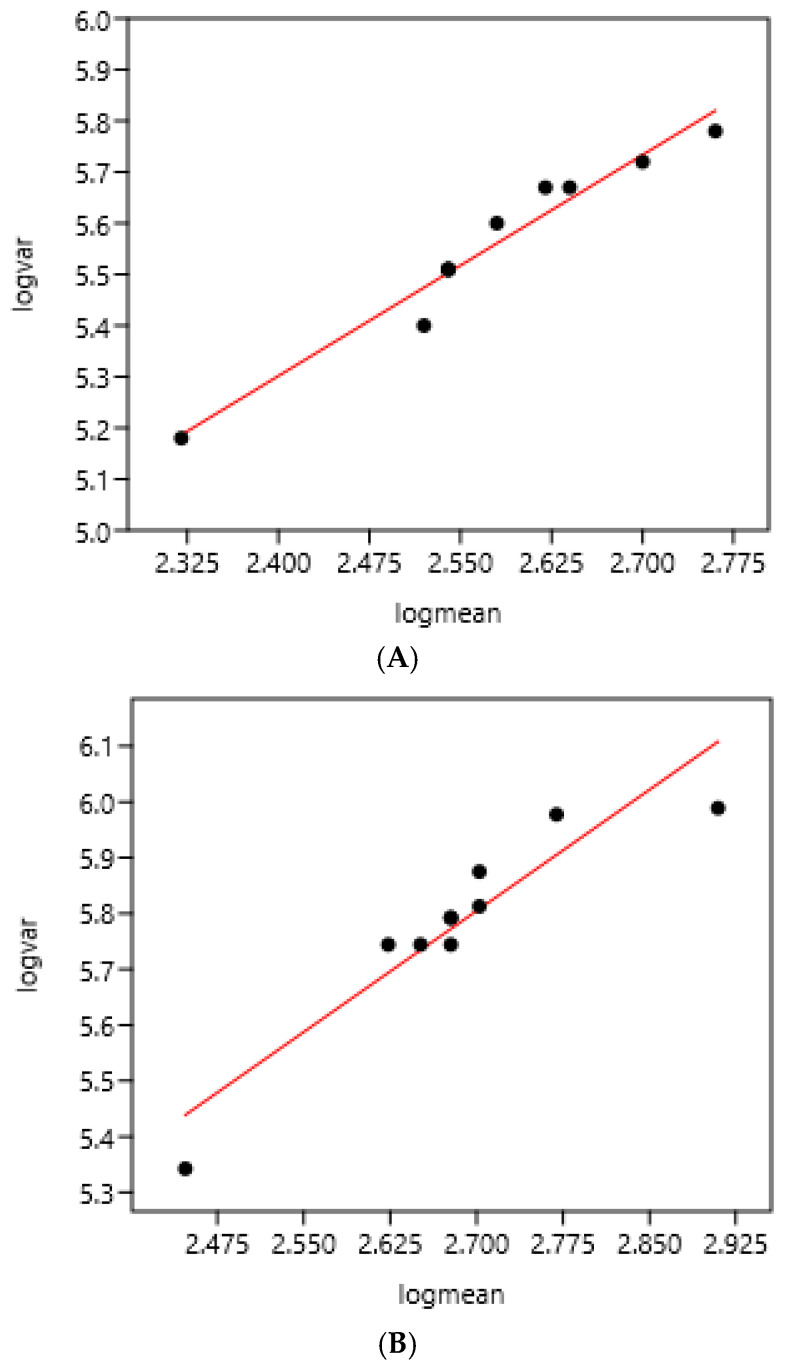
Relation between variance and mean of EPG on two consecutive days of faecal sampling in a farm (France 1) using 10 bootstrap samples each day (43 horses). (**A**) Faecal sampling on day 1: log (var) = 1.85 + 1.43 log (mean) r = 0.98 n = 10 (mean = 391; standard deviation = 642). (**B**) Faecal sampling on day 2: log (var) = 1.90+ 1.45 log (mean) r = 0.93 n = 10 (mean = 465; standard deviation = 777).

There was a significant relationship between variance and mean on the two consecutive days of faecal sampling (Figure 1). The slope coefficients of the regression were highly similar: 1.43 and 1.45.

**Figure 2:**
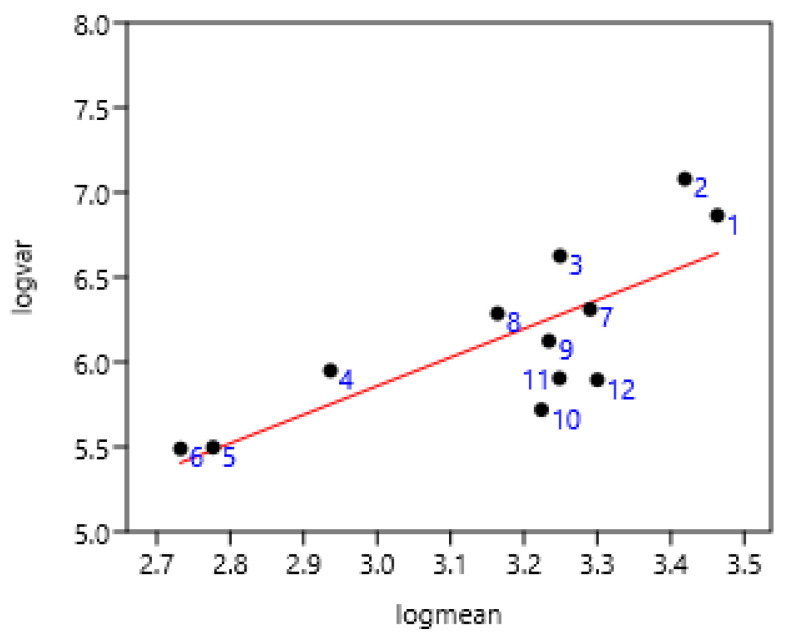
Relationship between FEC variance and mean at seven-day intervals on Mexican farms in four regions (Mexico 1: 1, 2, 3; Mexico 2: 4, 5, 6; Mexico 3: 7, 8, 9; Mexico 4: 10, 11, 12) (261 horses). log (var) = 0.80 + 1.69 log (mean); r = 077 *p* = 0.003.

A significant relationship between FEC variance and mean was observed on four farms of Mexico sampled at seven-day intervals (Figure 2). There were slight differences between Mexico 1 and Mexico 4 compared to others, probably due to climatic conditions.

#### 3.1.2. Age of Horses

**Figure 3:**
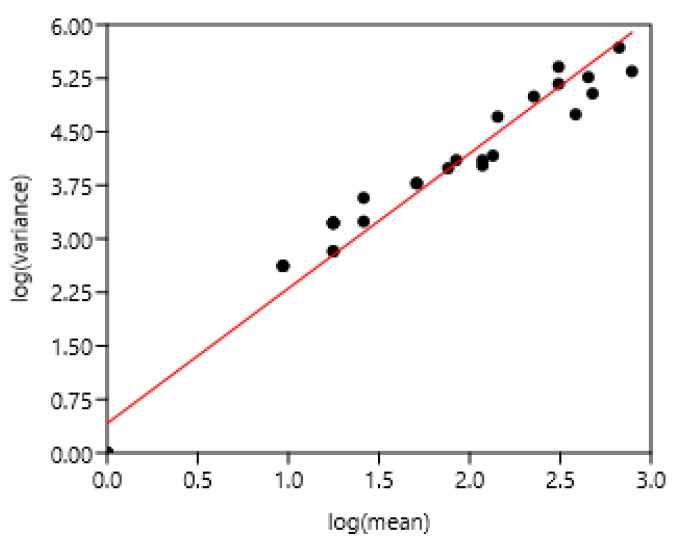
Relation between FEC variance and mean in Welsh ponies from 2 to 9 years old on six occasions during two years of sampling. Each point corresponds to values concerning one age at different periods of sampling. log (variance) = 0.41 + 1.89 log (mean) r = 0.98, n = 32, *p* < 0.0001.

A significant relationship between FEC and its variability among different ages of ponies (2 to 9 years old) did also exist in Welsh ponies of the France 1 farm (Figure 3).

#### 3.1.3. Category of Horses

**Figure 4:**
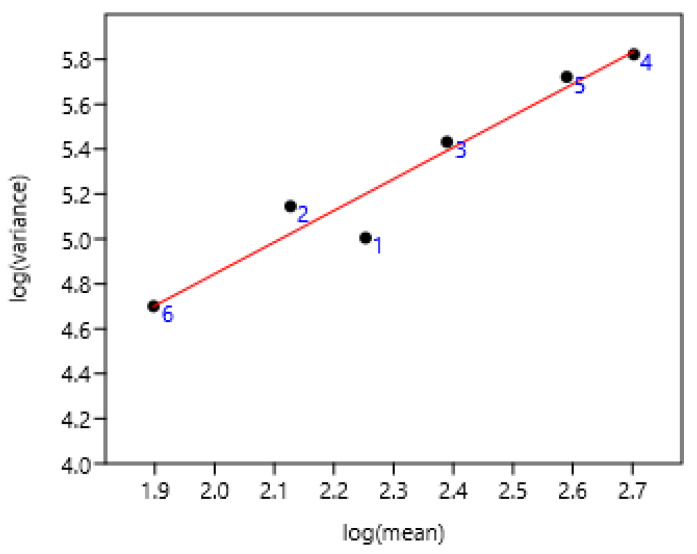
Relationship between variance and mean among different categories of horses in Poland. 1 Gelding (n = 281), 2 stallion (n = 2072), 3 mare (n = 6648), 4 two years (n = 1379), 5 yearling (n = 1865), 6 foal (n = 357). log (var) = 2.03 + 1.41 log (mean); r = 0.97 n = 6 *p* = 0.001.

This was established on all the Poland data. Geldings and stallions were the most differing from the linear regression (Figure 4). They had reduced access to grazing compared to other categories.

#### 3.1.4. Regions of Three Countries

**Figure 5:**
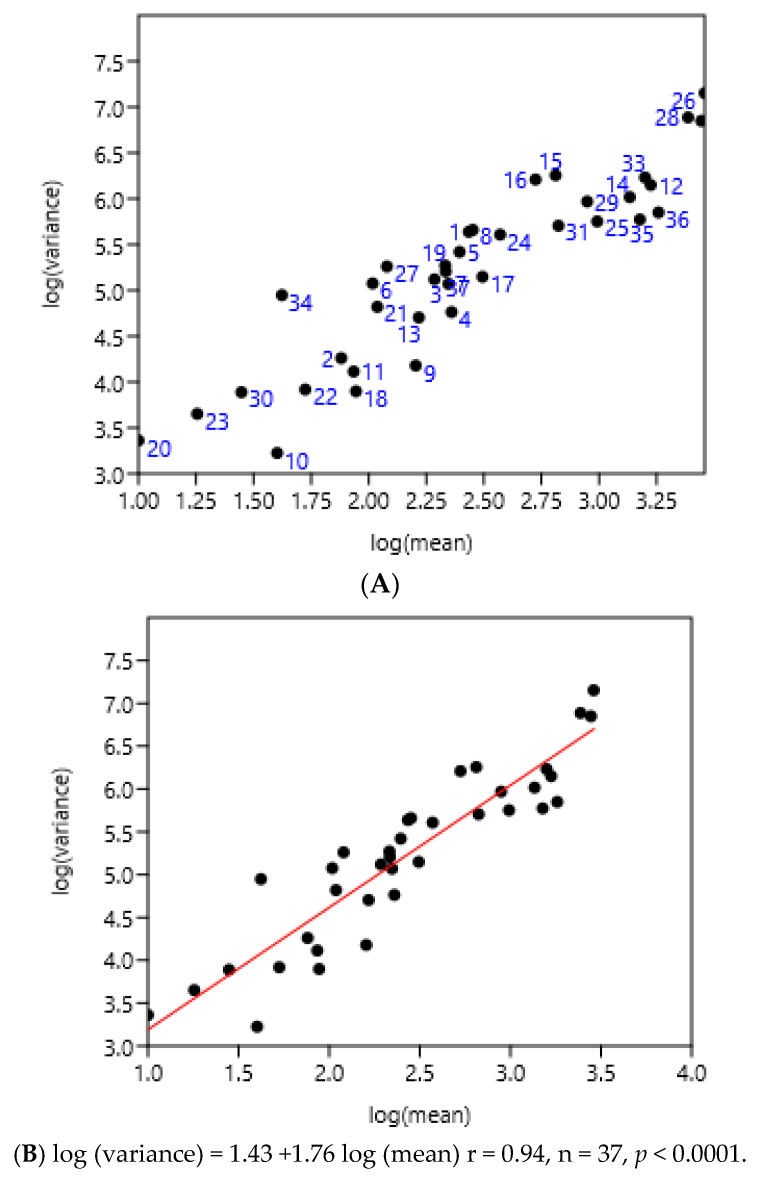
Relationship between variance and mean among horses and ponies of three countries. (**A**) 1–8 Poland, 9–25 France, 26–37 Mexico, (**B**) linear regression.

The entire set of data was used. There was a highly significant relationship between the variance and mean of FEC (Figure 5). There was no effect of region or country.

#### 3.1.5. Previous Anthelmintic Treatment

**Figure 6:**
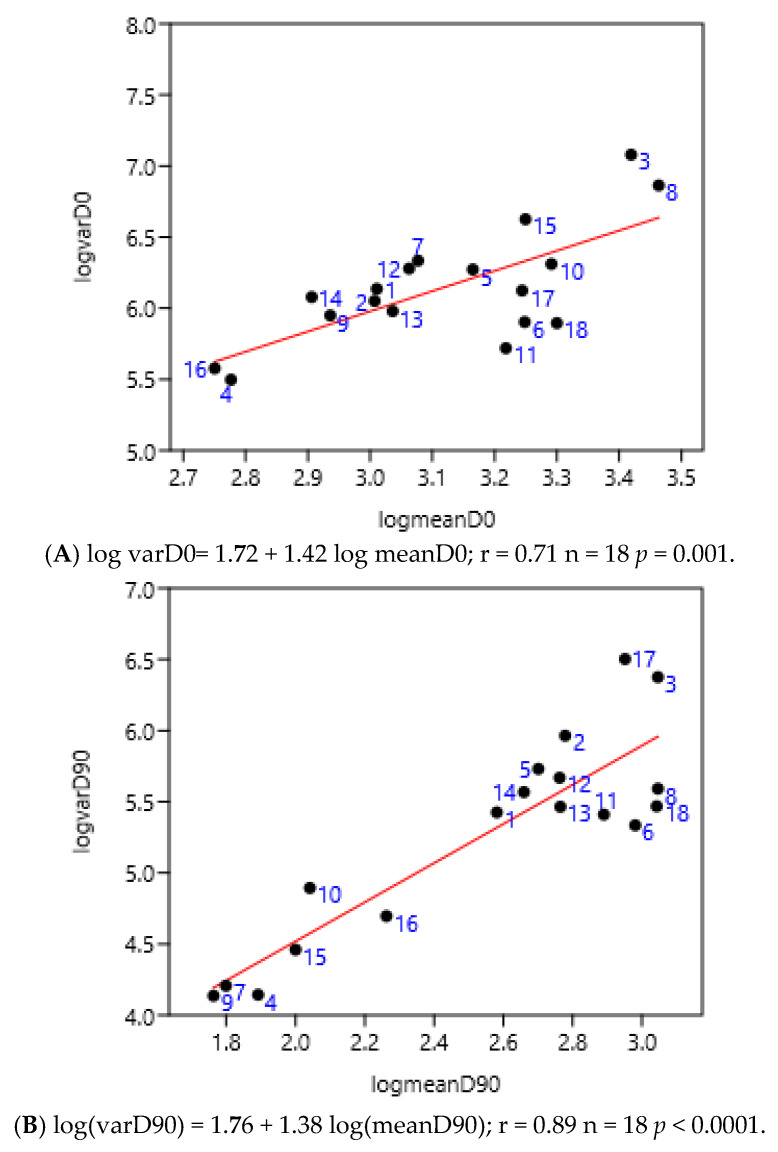
Relationship between variance and mean among horses (France 2; Mexico 1, 2, 3, 4) and ponies (France 1) before (**A**) and 90 days after (**B**) anthelmintic treatment (moxidectin 1–6, ivermectin 7–11, pyrantel 12–13, benzimidazoles 14–18).

There was no significant difference between before and after 90 after treatment days (Figure 6A,B). The numbers corresponded to the different anthelmintic treatments in the farms. The positions of farms treated with benzimidazoles (from 14 to 18), for example, were not grouped either before or after treatment. The treatment with different anthelmintics did not influence the value of the slope of the linear regression of variance to mean: 1.38 vs. 1.42.

### 3.2. Composite Faecal Egg Count to Establish Average FEC

Composite FEC may be established from faeces collected individually from horses or sampling on the ground of the stable.

#### 3.2.1. Composite from Faeces Collected from Horses

**Figure 7:**
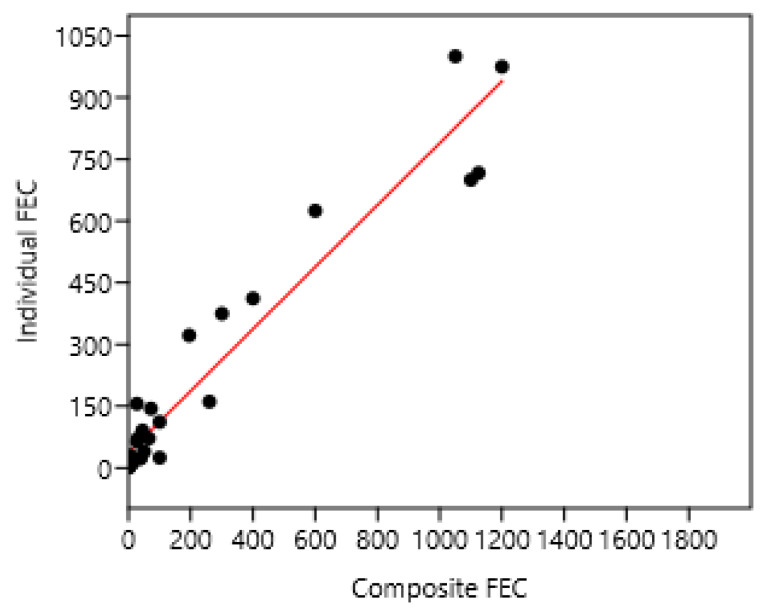
Relation between mean composite and individual faecal egg counts (FECs) in France 1 and 3. A regression on logarithm transformed data was also significant: log (individual FEC) = 0.82 + 0.42 log (composite FEC) n = 28; r = 0.95; *p* < 0.001. Individual FEC = 37 + 0.75; composite FEC n = 28 r = 0.96 *p* < 0.001.

Ten composite samples were examined and compared to 10 individual horse samples in France 1 and 3 (Figure 7).

#### 3.2.2. Composite from Faeces Collected from the Ground

Mean composite and individual FEC evaluations (France 1) based on 10 samples were significantly correlated (r_s_ = 0.80; *p* = 0.001). A significant regression was established between the two evaluations of mean FEC:

log (individual FEC) = 0.75 + 0.72 log (composite FEC) n = 12; r = 0.92; *p* < 0.001.

### 3.3. Relation Between Mean FEC and the Percentage of Horses with Different Indicators of FEC

**Figure 8:**
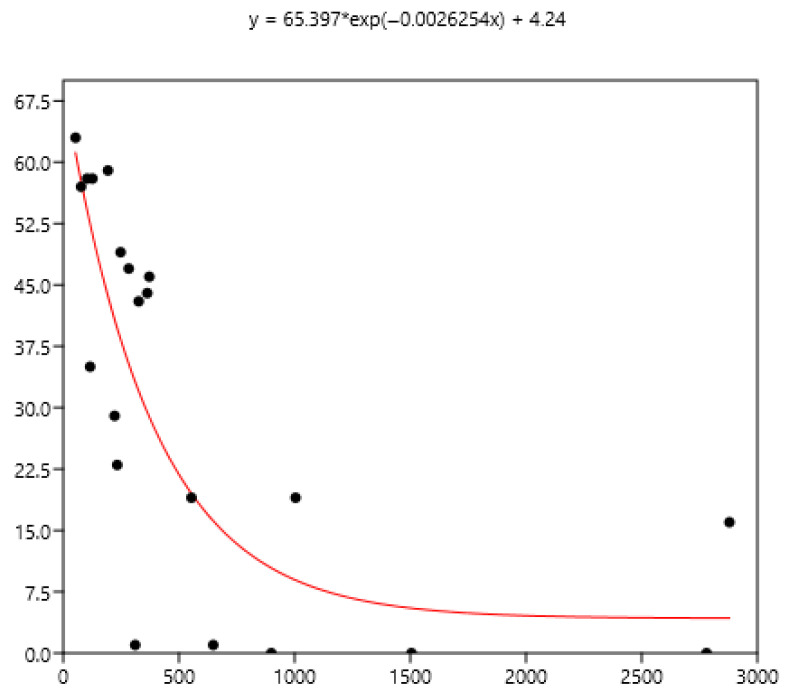
Exponential relationship between FEC mean (x) of a group of horses and percentage of FEC = 0 counts (y).

**Figure 9:**
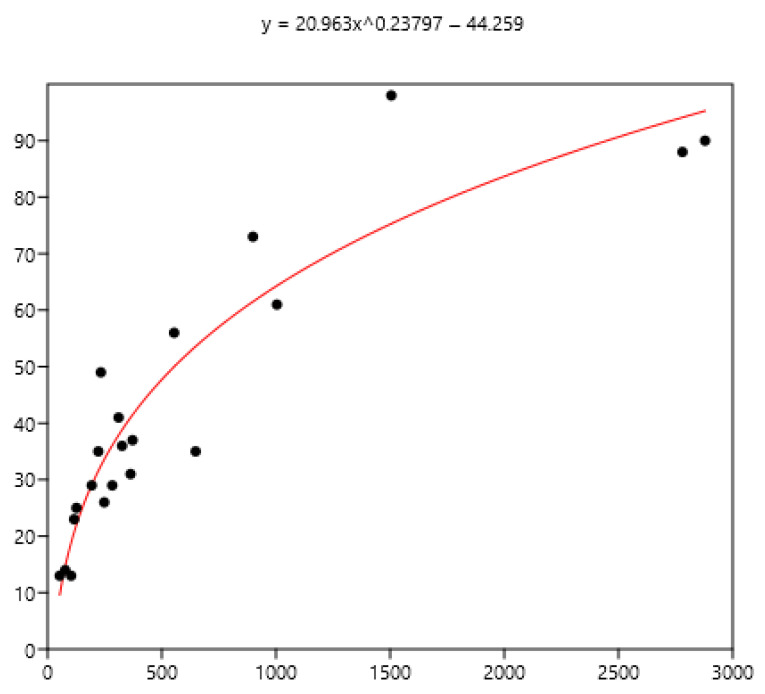
Power relationship between FEC mean (x) of a group of horses and percentage of FEC counts over 200 (y).

**Figure 10:**
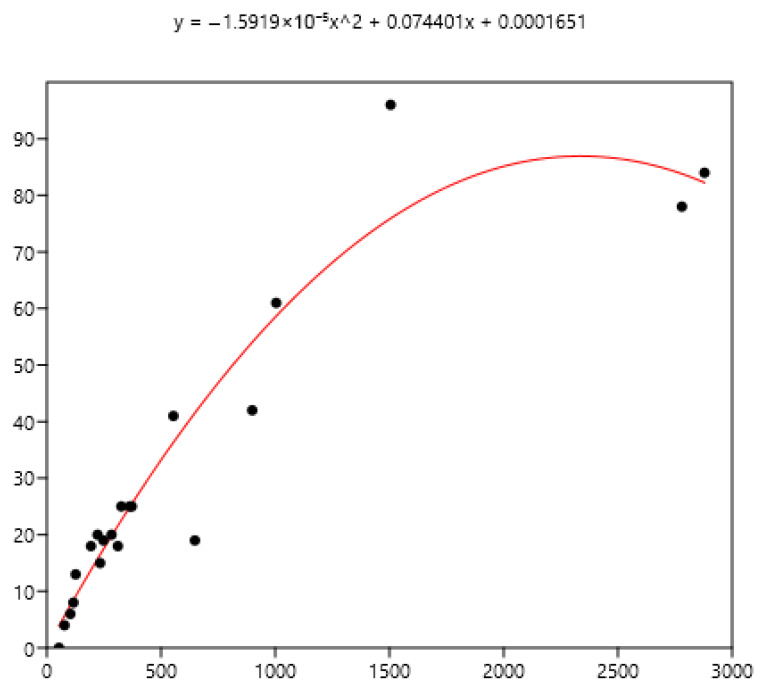
Power relationship between FEC mean (x) of a group of horses and percentage of FEC counts over 400 (y).

The mean FEC is related significantly (r_s_) to variance (0.79), FEC = 0 (−0.80), FEC < 100 (−0.86), FEC > 200 (0.90), and FEC > 400 (0.94) using the data set of the three countries. The non-linear fit was performed between the mean and the different percentages of FECs. The equations are in Figure 8, Figure 9 and Figure 10.

The mean is the least related (and negatively) to the percentages of FEC = 0 (Figure 8). The mean is positively related to FEC over 200 or 400 and linear up to a mean FEC of 1000 EPG. Thus, in Figure 9, a mean of 1000 indicates that 65% of the horses should be treated and only 35% for a mean FEC of 250. If we choose the cut-off of 400 EPG (Figure 10), then when the mean FEC is 1000, 55% of the animals should be treated, and only 15% for a mean FEC of 250. When the mean FEC is over 1500, most of the horses should be treated.

## 4. Discussion

The tendency of population density to vary as a power function of the mean was first observed in the field of ecology [8]. Since then, this concept has been extended to all complex systems, including physics (where it is known as fluctuating scaling), climatology, the stock market, and the life sciences [32]. Adult parasite species and their propagules interact with each other and their hosts in complex ways [33]. TPL has been found applicable to fecundity (FEC) in horses’ GIN, as it was to adult GIN in sheep [7] and other ruminants [11]. In the latter study, TPL was evaluated in the FEC of only two species (*Nematodirus* and *Marshallagia)*, which have easily identifiable eggs. The slope of the regression of variance to mean was shown to be similar for FEC data and adult parasites with *Marshallagia* but lower with *Nematodirus*. While there are probably some differences between FEC and adult parasite counts, these are not very significant; the slope values were close to 2 for sheep [7], 1.7 in several species of ruminant [11], and 1.76 for our current FEC in horses.

Despite there being empirical illustrations in various areas of parasitism, the biological significance of this relationship is still being debated. It is likely that the combined effects of biological and statistical processes on parasite aggregation influence the TPL [33]. The slope of the TPL clearly defines the relationship between the mean and variance. Taylor [8] suggested that it is also an index of aggregation that describes an intrinsic property of the organisms in question. However, it is unclear how the TPL slope relates to other aggregation concepts, such as k, the variance-to-mean ratio, the Gini index, and the Hoover index [34]. Therefore, it is difficult to relate explanations based on aggregation indices to variations in the TPL slope. In another host–parasite model [35], the results suggested that host factors alone, operating post-infection, were sufficient to generate strong aggregation of parasite distributions rather than heterogeneity in exposure and initial invasion. General modelling also indicated that the host’s immune protective response generates aggregation [36]. Boag et al. [37] also proposed that host immunity plays a significant role in determining the TPL slope in natural gastrointestinal helminth infections in rabbits. However, this was not observed in our data because the slope did not change with age (Figure 2) or the horses’ status (Figure 3). The slope of TPL was not altered by the presence of large strongyles (Mexico data), even when mean FECs were higher. The lack of variability in the slope of TPL indicates that the proportion of highly excreting horses in a group can be deduced from the mean (Figure 8 and Figure 9), which is useful for deciding whether to perform TST.

However, farmers are not motivated to use FEC due to the comparative cost of treatment and FEC [25], as well as the time required for faecal sampling. The decision to use FEC is not influenced by the perceived risk of anthelmintic resistance or GIN infection but is influenced by a negative attitude towards anthelmintics and a desire to control deworming programmes, which is associated with an increase in social pressure [26]. Using mean FEC with pooled (composite) faeces could reduce the number of laboratory FEC tests required and hence the cost. Previous studies have examined composite FECs in sheep [38] and different categories of horses [39]. In our study, composite evaluation was efficient on a sample of 10 composite FECs based on faeces from 10 individuals. Sampling from horses is time-consuming, so composite sampling based on faeces collected from the stable floor is an interesting procedure to propose to farmers as it eliminates the need for time-consuming individual sampling.

The decision to use TST depends on the type of horse; it should not be used for foals, who are sensitive to parasites [40]. The exclusion of foals is not always accepted, and TST is also applied to them [41] since TST is intended to cope with anthelmintic resistance and it concerns all nematodes of all horses [42]. It also depends on the group of parasites: the use of TST favours the maintenance of large strongyles [24] (odds ratio 4.4), and their presence in our Mexican data may preclude its careless use. Even when all the necessary conditions are met, the chosen mean cut-off, ranging from 200 to 400 FEC [15] or even 500 FEC [40,43], may affect its effectiveness. However, when over 50% of horses need treatment, the interest in TST is limited (mean EPG 500 with a cut-off of 200, or mean EPG 750 with a cut-off 400: Figure 8 and Figure 9). When needed, the choice of animals to be treated can focus on risk groups, such as horses up to five or six years old [42,44], excluding foals, according to [42], that should not be submitted to TST. This selection could also be based more accurately on individual FEC. However, it should be borne in mind that these FECs can vary [45], and substantial variation can be seen between samples taken from the same animal [46], even if 94% exhibit consistent egg shedding [47]. The main objective is to minimize the number of treatments in order to reduce the development of anthelmintic resistance while treating those with a higher level of contamination, i.e., above 200 EPG [20]. However, EPGs are not an exact reflection of actual adult infection; above 500 EPGs, the level is poorly related to the number of worms [43]. Worm fertility varies considerably [48], which may affect interpretation for clinical use. Equines excrete 8 g of dry faecal matter per kg of body weight [49], so the same FEC in a Welsh pony weighing 300 kg and a Thoroughbred weighing 500 kg does not have the same meaning in terms of worm infection. This could partly influence the cut-off values in relation to the possible clinical impact of GIN. Further research is needed to adapt these values according to farm structures to reduce the development of resistance and limit clinical outcomes. It should also be borne in mind that anthelmintic treatment has to be chosen in agreement with gastrointestinal parasites including pinworms, *Parascaris*, tapeworms, and *Gasterophilidae* and that all parasite infections should be considered. When small strongyles are the main problem, TST can be undertaken with confidence, but the use of anthelmintics is not the only means to control GIN and good grazing management should be associated with TST [39].

## 5. Conclusions

The TPL is an efficient way of relating the mean and variation in the FECs (small and large strongyles) of horses. The percentage of horses with a higher FEC than a predetermined threshold can be easily calculated from the mean FEC and used to inform selective anthelmintic treatment. The remaining technical problems are choosing a cut-off point and which horses should be treated. No treatment is required when the mean FEC is below the cut-off point. If treatment is required, it may be necessary to focus on horses known to be at risk due to their age, previous identification, or clinical status, which may be associated with gastrointestinal nematodes. Repeated use of targeted selective treatment should be carried out with caution when large strongyles are present.

## Figures and Tables

**Table 1:** Characteristics of the studied farms.

Country	Climate on Yearly Basis Rainfall in mm (Temperature)	Horse Breed	Type of Horse (Number)	Number of Farms/Duration in Years	Anthelmintic Treatments	Anthelmintic Efficacy (%)	Percentage of Cyathostomins
Poland (South-East Part)	680 (8.7 °C)	Pure-Blood Arabian, Thoroughbred	Racing (12,450 altogether)	74 (10)	Pyrantel, Fenbendazole Ivermectin Moxidectin	51 * 23 67 100	99
France 1	689 (11.8 °C)	Welsh Pony	Leisure (100 × 10 sampling events)	1 (3)	Ivermectin, Moxidectin Pyrantel	90 100 90	98
France 2	579 (14.8 °C)	Diverse	Riding club (35 × 4 sampling events)	1 (0.5)	Ivermectin, Mebendazole	96 76	Nd **
France 3	1237 (10.8 °C)	Selle Français, Anglo-Arab	Racing (160 × 6 sampling events)	1 (2)	Ivermectin, Pyrantel Fenbendazole	96 97 55	95
Mexico 1	1240 (25.0 °C)	American Quarter	Racing (42 × 4 sampling events)	1 (0.25)	Ivermectin, Moxidectin, Febantel	99 90 97	64
Mexico 2	490 (24.0 °C)	American Quarter	Racing (54 × 4 sampling events)	1 (0.25)	Ivermectin, Moxidectin, Febantel	99 98 90	50
Mexico 3	426 (22.5 °C)	American Quarter	Racing (71 × 4 sampling events)	1 (0.25)	Ivermectin, Moxidectin, Febantel and Metrifonate	100 100 93	30
Mexico 4	747 (26.5 °C)	Raza Española	Racing (99 × 4 sampling events)	1 (0.25)	Ivermectin, Moxidectin, Febantel and Metrifonate	100 100 100	65

* These efficacies were calculated in comparison with moxidectin. Others were calculated with untreated control according to [29]. ** Not determined.

## Data Availability

Data are available within this paper.
